# Supplementary material for: Low-Temperature Predicted Structures of Ag2S (Silver Sulfide)
Source: Nanomaterials (Basel). 2023 Sep 25;13(19):2638. doi: 10.3390/nano13192638 (PMC10574637; doi:10.3390/nano13192638)
Supplement: Supplementary file 1 [file nanomaterials-13-02638-s001.zip › nanomaterials-2595278-supplementary.pdf]

Nanomaterials

Supplementary Material

Low-Temperature Predicted Structures of Ag_2S Silver Sulfide

S. I. Sadovnikov[1], M. G. Kostenko[1], A. I. Gusev[*1], A. V. Lukoyanov[2,3]

[1] Institute of Solid State Chemistry, Ural Branch of the Russian Academy of Sciences, 620990 Ekaterinburg, Russia; sadovnikov@ihim.uran.ru (SS); makskostenko@yandex.ru (MK); gusev@ihim.uran.ru (AG)

[2] Mikheev Institute of Metal Physics, Ural Branch of the Russian Academy of Sciences, 20108 Ekaterinburg, Russia; lukoyanov@imp.uran.ru (AL)

[3] Ural Federal University named after the first President of Russia B. N. Yeltsin, 620002 Ekaterinburg, Russia; lukoyanov@imp.uran.ru (AL)

E-mail: gusev@ihim.uran.ru; Tel.: +08-343-3747306

[*] Corresponding author. Tel.: +7 343 374 7306; fax: +7 343 374 4495.
E-mail address: gusev@ihim.uran.ru (A.I. Gusev).

Table S1

Energies E_{VASP} of individual Ag and S atoms and the condensed metallic silver Ag and condensed sulfur S phase

Atoms Ag and S				Condensed Ag (space group No.225 - $Fm\bar{3}m$) and S (space group No,70 - *Fddd*) phases			
number of atoms		* E_{VASP} (eV)		number of atoms		* E_{VASP} (eV)	
$N_{Ag\text{-}at}$	$N_{S\text{-}at}$	$E_{Ag\text{-}at}$	$E_{S\text{-}at}$	$N_{Ag\text{-}cond}$	$N_{S\text{-}cond}$	$E_{Ag\text{-}cond}$	$E_{S\text{-}cond}$
1	1	-0.1982	-1.0808	4	128	-10.8628	-528.1684

* energies E_{VASP} are obtained by the DFT calculations.

Table S2

Model predicted cubic Ag_2S structures

Space group	Atom	Position and multiplicity	Atomic coordinates in the model structures		
			$x/a \equiv x/a_{cub}$	$y/b \equiv y/b_{cub}$	$z/c \equiv z/c_{cub}$
*No.224 - $Pn\bar{3}m$	Ag	4(*b*)	0.2500001	0.2500001	0.2500001
	S	2(*a*)	0	0	0
**No.227 - $Fd\bar{3}m$	Ag	32(*e*)	0.0800106	0.0800106	0.0800106
	S1	8(*a*)	0	0	0
	S2	8(*b*)	0.5	0.5	0.5

* parameters of the predicted unit cell (space group $Pn\bar{3}m$): $a = b = c = 0.544009$ nm, $Z = 2$, $V = 0.16099736$ nm^3, **a** = [100]$_{cub}$, **b** = [010]$_{cub}$, and **c** = [001]$_{cub}$;
** parameters of the predicted unit cell (space group $Fd\bar{3}m$): $a = b = c = 1.723559$ nm, $Z = 8$, $V = 5.120101924$ nm^3, **a** = [100]$_{cub}$, **b** = [010]$_{cub}$, and **c** = [001]$_{cub}$.

Table S3

Model predicted tetragonal Ag_2S structures

Space group	Atom	Position and multiplicity	Atomic coordinates in the model structures		
			$x/a \equiv x/a_{tetr}$	$y/b \equiv y/b_{tetr}$	$z/c \equiv z/c_{tetr}$
*No.116 - $P\bar{4}c2$	Ag1	4(*e*)	0.2303562	0.2303562	0.25
	Ag2	4(*f*)	0.2303562	0.2303562	0.75
	S1	2(*b*)	0.5	0.5	0.25
	S2	2(*c*)	0	0	0
**No.123 - *P*4/*mmm*	Ag1	1(*a*)	0	0	0
	Ag2	1(*b*)	0	0	0.5
	S	1(*d*)	0.5	0.5	0.5

* parameters of the predicted unit cell (space group $P\bar{4}c2$): $a = b = 0.628234$ nm, $c = 0.715433$ nm), $Z = 4$, $V = 0.282365157$ nm^3, **a** = [100]$_{tetr}$, **b** = [010]$_{tetr}$, and **c** = [001]$_{tetr}$;
** parameters of the predicted unit cell (space group *P*4/*mmm*): $a = b = 0.357475$ nm, $c = 0.546104$ nm), $Z = 1$, $V = 0.069785624$ nm^3, **a** = [100]$_{tetr}$, **b** = [010]$_{tetr}$, and **c** = [001]$_{tetr}$.

Table S4

Model predicted trigonal Ag_2S structures

Space group	Atom	Position and multiplicity	Atomic coordinates in the model structures		
			$x/a \equiv x/a_{trig}$	$y/b \equiv y/b_{trig}$	$z/c \equiv z/c_{trig}$
*No.148 - $R\bar{3}$	Ag	2(*c*)	0.2500007	0.2500007	0.2500007
	S	1(*a*)	0	0	0
**No. 166 - $R\bar{3}m$	Ag1	2(*c*)	0.1249771	0.1249771	0.1249771
	Ag2	2(*c*)	0.3750229	0.3750229	0.3750229
	S1	1(*a*)	0	0	0
	S2	1(*b*)	0.5	0.5	0.5

* parameters of the predicted unit cell (space group $R\bar{3}$): $a = b = c = 0.440645$ nm, $\alpha = 60.005°$, $V = 0.060506592$ nm^3, $Z = 1$;

** parameters of the predicted unit cell (space group $R\bar{3}m$): $a = b = c = 0.763092$ nm, $\alpha = 33.5637°$, $V = 0.120980424$ nm^3, $Z = 2$.

Table S5

Ag – S bond lengths for the predicted Ag_2S structures with different symmetry

Symmetry and space group	Bond	
	type	length (nm)
Cubic (No.224 - $Pn\bar{3}m$)	Ag - S	0.23556
Cubic (No.227 - $Fd\bar{3}m$)	Ag – S1	0.23883
	Ag – S2	0.65451
Tetragonal (No.116 - $P\bar{4}c2$)	Ag1 – S1	0.23957
	Ag1 – S2	0.27180
	Ag2 – S1	0.23957
	Ag2 – S2	0.27180
Tetragonal (No.123 - $P4/mmm$)	Ag1 – S	0.37209
	Ag2 – S	0.25277
Trigonal (No.148 - $R\bar{3}$)	Ag - S	0.23174
Trigonal (No.166 - $R\bar{3}m$)	Ag1 – S1	0.25749
	Ag2 – S2	0.25749
Orthorhombic (No.64 - *Cmce*)	Ag1 – S	0.24306
	Ag2 – S	0.24357
Orthorhombic (No.63 – *Cmcm*)	Ag1 – S	0.24033
	Ag2 – S	0.25233
Orthorhombic (No.36 - $Cmc2_1$)	Ag - S	0.25232
Orthorhombic (No.19 - $P2_12_12_1$)	Ag1 – S	0.25682
	Ag2 – S	0.26311
Unrelaxed monoclinic (No.14 - $P2_1/c$) acanthite	Ag1 – S	0.25113
	Ag2 – S	0.25475
Relaxed monoclinic (No.14 - $P2_1/c$) acanthite	Ag1 – S	0.25242
	Ag2 – S	0.24030
Monoclinic (No.14 - $P2_1/c$)	Ag1 – S	0.24177
	Ag2 – S	0.24281
Triclinic (No.1 - *P*1)	Ag1 – S1	0.24962
	Ag1 – S3	0.24976
	Ag2 – S2	0.24718
	Ag2 – S4	0.24756
	Ag3 – S2	0.24943
	Ag3 – S4	0.24891
	Ag4 – S1	0.24374
	Ag4 – S4	0.24388
	Ag5 – S1	0.24401
	Ag5 – S4	0.24400
	Ag6 – S1	0.24723
	Ag6 – S3	0.24804
	Ag7 – S2	0.24417
	Ag7 – S3	0.24388
	Ag8 – S2	0.24373
	Ag8 – S3	0.24395

Matrices of elastic stiffness constants computed for model Ag_2S structures

$$\boldsymbol{C}_{\text{cubic } Pn\text{-}3m} = \begin{pmatrix} 46.1554 & 43.2273 & 43.2273 & 0 & 0 & 0 \\ 43.2273 & 46.1554 & 43.2273 & 0 & 0 & 0 \\ 43.2273 & 43.2273 & 46.1554 & 0 & 0 & 0 \\ 0 & 0 & 0 & 1.1875 & 0 & 0 \\ 0 & 0 & 0 & 0 & 1.1875 & 0 \\ 0 & 0 & 0 & 0 & 0 & 1.1875 \end{pmatrix}, \qquad \text{(S6)}$$

$$\boldsymbol{C}_{\text{cubic } Fd\text{-}3m} = \begin{pmatrix} 5.8029 & 5.3912 & 5.3912 & 0 & 0 & 0 \\ 5.3912 & 5.8029 & 5.3912 & 0 & 0 & 0 \\ 5.3912 & 5.3912 & 5.8029 & 0 & 0 & 0 \\ 0 & 0 & 0 & 0.8795 & 0 & 0 \\ 0 & 0 & 0 & 0 & 0.8795 & 0 \\ 0 & 0 & 0 & 0 & 0 & 0.8795 \end{pmatrix}, \qquad \text{(S7)}$$

$$\boldsymbol{C}_{\text{tetr } P\text{-}4c2} = \begin{pmatrix} 77.3256 & 80.6809 & 178.5539 & 0 & 0 & 0 \\ 80.6809 & 77.3256 & 24.4436 & 0 & 0 & 0 \\ 24.4436 & 24.4436 & 46.9722 & 0 & 0 & 0 \\ 0 & 0 & 0 & 7.1893 & 0 & 0 \\ 0 & 0 & 0 & 0 & 7.1893 & 0 \\ 0 & 0 & 0 & 0 & 0 & 31.5106 \end{pmatrix}. \qquad \text{(S8)}$$

$$\boldsymbol{C}_{\text{tetr } P4/mmm} = \begin{pmatrix} 93.0592 & 77.4578 & 9.1840 & 0 & 0 & 0 \\ 77.4578 & 93.0592 & 9.1840 & 0 & 0 & 0 \\ 9.1840 & 9.1840 & 112.6081 & 0 & 0 & 0 \\ 0 & 0 & 0 & -9.5228 & 0 & 0 \\ 0 & 0 & 0 & 0 & -9.5228 & 0 \\ 0 & 0 & 0 & 0 & 0 & 30.1880 \end{pmatrix}, \qquad \text{(S9)}$$

$$\boldsymbol{C}_{\text{trig } R\text{-}3} = \begin{pmatrix} 84.3399 & 54.8023 & 54.9045 & 2.8741 & -1.1961 & -1.1750 \\ 54.8023 & 84.2519 & 55.0124 & -1.0602 & 2.9786 & -1.3465 \\ 54.9045 & 55.0124 & 84.1708 & -1.2232 & -1.2021 & 3.0918 \\ 2.8741 & -1.0602 & -1.2232 & 10.0111 & 2.6086 & 2.4867 \\ -1.1961 & 2.9786 & -1.2021 & 2.6086 & 9.9200 & 2.3735 \\ -1.1750 & -1.3465 & 3.0918 & 2.4867 & 2.3735 & 9.8357 \end{pmatrix}, \qquad \text{(S10)}$$

$$\boldsymbol{C}_{\text{trig } R\text{-}3m} = \begin{pmatrix} 76.4621 & 58.0645 & 58.7782 & -1.0093 & 2.4718 & 3.4956 \\ 58.0645 & 76.7396 & 59.8201 & 2.8060 & 0.6403 & -3.7360 \\ 58.7782 & 59.8201 & 76.3559 & -2.2633 & -3.9201 & -0.9024 \\ -1.0093 & 2.8060 & -2.2633 & 14.6368 & -0.5129 & 0.9157 \\ 2.4718 & 0.6403 & -3.9201 & -0.5129 & 14.0446 & -0.8503 \\ 3.4956 & -3.7360 & -0.9024 & 0.9157 & -0.8503 & 13.4434 \end{pmatrix}, \quad \text{(S11)}$$

$$\boldsymbol{C}_{\text{orthorhomb } Cmcm} = \begin{pmatrix} 98.4970 & 38.3156 & 22.4833 & 0 & 0 & 0 \\ 38.3156 & 44.7754 & 10.0698 & 0 & 0 & 0 \\ 22.4833 & 10.0698 & 29.2198 & 0 & 0 & 0 \\ 0 & 0 & 0 & 4.4577 & 0 & 0 \\ 0 & 0 & 0 & 0 & 12.5734 & 0 \\ 0 & 0 & 0 & 0 & 0 & 8.1594 \end{pmatrix}, \quad \text{(S12)}$$

$$\boldsymbol{C}_{\text{orthorhomb } Cmce} = \begin{pmatrix} 65.3687 & 5.0551 & 4.3304 & 0 & 0 & 0 \\ 5.0551 & 57.2074 & 4.3660 & 0 & 0 & 0 \\ 4.3304 & 4.3660 & 11.8096 & 0 & 0 & 0 \\ 0 & 0 & 0 & 1.5834 & 0 & 0 \\ 0 & 0 & 0 & 0 & 1.8333 & 0 \\ 0 & 0 & 0 & 0 & 0 & 21.6154 \end{pmatrix}, \quad \text{(S13)}$$

$$\boldsymbol{C}_{\text{mon } P21} = \begin{pmatrix} 11.5680 & -0.5756 & 3.5408 & 0 & -2.3344 & 0 \\ -0.5756 & 44.8670 & 8.2904 & 0 & -4.5026 & 0 \\ 3.5408 & 8.2904 & 44.6176 & 0 & -10.5227 & 0 \\ 0 & 0 & 0 & 31.1857 & 0 & -2.9951 \\ -2.3344 & -4.5026 & -10.5227 & 0 & 2.4748 & 0 \\ 0 & 0 & 0 & -2.9951 & 0 & 3.5431 \end{pmatrix}, \quad \text{(S14)}$$

$$\boldsymbol{C}_{\text{mon unrelax } P21/c} = \begin{pmatrix} 57.6611 & 19.1243 & 40.2312 & 0 & -4.3388 & 0 \\ 19.1243 & 32.2911 & 18.1162 & 0 & 0.8730 & 0 \\ 40.2312 & 18.1162 & 64.2186 & 0 & -14.1836 & 0 \\ 0 & 0 & 0 & 7.0640 & 0 & -1.8039 \\ -4.33388 & 0.8730 & -14.4836 & 0 & 18.8466 & 0 \\ 0 & 0 & 0 & -1.8039 & 0 & 6.6861 \end{pmatrix}, \quad \text{(S15)}$$

$$\boldsymbol{C}_{\text{mon relax } P21/c} = \begin{pmatrix} 53.8604 & 13.9430 & 46.1417 & 0 & -9.1408 & 0 \\ 13.9430 & 29.2109 & 19.1649 & 0 & -6.0901 & 0 \\ 46.1417 & 19.1649 & 74.1809 & 0 & -15.0413 & 0 \\ 0 & 0 & 0 & 9.9337 & 0 & -3.5613 \\ -9.1408 & -6.0901 & -15.0413 & 0 & 14.4149 & 0 \\ 0 & 0 & 0 & -3.5613 & 0 & 6.7978 \end{pmatrix}, \quad \text{(S16)}$$

$$\boldsymbol{C}_{\text{tricl } P1} = \begin{pmatrix} 72.5669 & 20.5727 & 24.8878 & -0.0539 & -3.5796 & 1.1041 \\ 20.5727 & 36.9730 & 19.3270 & -0.2075 & -1.0575 & 0.7500 \\ 24.8878 & 19.3270 & 37.2742 & -0.2435 & -3.4275 & 1.2591 \\ -0.0539 & -0.2075 & -0.2435 & 9.6464 & 0.7459 & -0.3204 \\ -3.5796 & -1.0575 & -3.4275 & 0.7459 & -0.7738 & -0.3785 \\ 1.1040 & 0.7500 & 1.2591 & -0.3204 & -0.3785 & 2.8872 \end{pmatrix}. \quad \text{(S17)}$$

The Voigt-Reuss-Hill averaging scheme [1].

Accordung to the Voigt-Reuss-Hill method, the Voigt and Reuss approximations lead to simplified relations between the polycrystalline constants and the single-crystal constants. For all crystal classes, these equations have the form:

$$B_V = [c_{11}+c_{22}+c_{33} + 2(c_{12} + c_{23} + c_{31})]/9, \quad \text{(S18}a\text{)}$$

$$B_R = 1/[s_{11} + s_{22} + s_{33} + 2(s_{12} + s_{23} + s_{31})], \quad \text{(S18}b\text{)}$$

$$G_V = [c_{11}+c_{22}+c_{33}+ 3(c_{44}+c_{55}+c_{66}) - (c_{12}+c_{23}+c_{31})]/15\ , \quad \text{(S18}c\text{)}$$

$$G_R = 15/[4(s_{11}+s_{22}+s_{33}) - 4(s_{12}+s_{23}+s_{31}) + 3(s_{44}+ s_{55}+ s_{66})]\ , \quad \text{(S18}d\text{)}$$

$$B_H = (B_V + B_R)/2,\ G_H = (G_V + G_R)/2. \quad \text{(S19)}$$

The elastic compliance constants s_{ij} are the coefficients of the inverted tensor of the elastic stiffness: $(\boldsymbol{S}) = (\boldsymbol{C})^{-1}$.

1. Hill, R. The elastic behaviour of a crystalline aggregate. *Proc. Phys. Soc. A*, **1952**, 65 (5), 349-354.
